# “Direct Me or Leave Me”: The Effect of Leadership Style on Stress and Self-Efficacy of Healthcare Professionals

**DOI:** 10.3390/bs15010025

**Published:** 2024-12-30

**Authors:** Stefan Milojević, Vesna Stojanović Aleksić, Marko Slavković

**Affiliations:** 1 Faculty of Business Economics, Educons University, Str. Vojvode Putnika 87, 21208 Sremska Kamenica, Serbia; stefan.milojevic@educons.edu.rs; 2 Faculty of Economics, University of Kragujevac, 34000 Kragujevac, Serbia; vesnasa@kg.ac.rs

**Keywords:** leadership, self-efficacy, employee well-being, stress, healthcare professionals

## Abstract

This study aims to investigate the influence of leadership on the self-efficacy of healthcare professionals. Additionally, it seeks to explore whether stress mediates the relationship between leadership and self-efficacy. Specifically, our study is focused on both transactional leadership and laissez-faire leadership, which are commonly practiced by healthcare professionals due to the settings of healthcare environments. This study utilized a structured questionnaire for measuring the leadership, stress, and self-efficacy of healthcare professionals. Data collection involved respondents rating these statements on a Likert scale. The sample consisted of 395 participants employed in healthcare organizations in Serbia. The analysis employed partial least squares structural equation modeling (PLS-SEM). The research findings indicate that laissez-faire leadership is positively associated with stress, while no significant negative impact on self-efficacy was observed. Transactional leadership did not demonstrate a significant relationship with reduced stress but was found to positively influence self-efficacy. Moreover, stress was identified as negatively impacting self-efficacy and mediated the association between laissez-faire leadership and self-efficacy, although no mediating effect was found for transactional leadership. This study underscores the critical role of leadership style in shaping the well-being and self-efficacy of healthcare professionals. By understanding how different leadership approaches impact employee stress and job satisfaction, healthcare organizations can tailor their management practices to foster a supportive work environment and enhance overall performance. The results emphasize the need for leaders to balance organizational objectives with employee needs, demonstrating effective communication and adaptability to promote a positive workplace culture.

## 1. Introduction

The efficiency of employees plays an important role in determining the level of employee energy invested and the initiative demonstrated by employees in their work (Ahmed et al., 2011). Self-efficacy, on the other hand, represents an individual’s assessment of their own capabilities and the ability to take specific actions to achieve goals. In essence, it reflects one’s perception of strengths and abilities that contribute to success in task completion (Waddington, 2023). Rooted in social cognitive theory, self-efficacy is linked to the belief that an individual can harness his or her capacities and cognitive resources to attain a desired outcome through action. As a dynamic construct, self-efficacy evolves with new experiences. It is closely related to self-esteem, yet differs in that self-esteem embodies a more intrinsic characteristic, whereas self-efficacy reflects a personal evaluation (Gist & Mitchell, 1992). Albert Bandura, the pioneer of self-efficacy theory, emphasizes that efficacy influences the level of effort individuals invest in tasks and the duration of those efforts. Higher self-efficacy corresponds to greater and more sustained efforts (Bandura, 1977). However, self-efficacy alone does not guarantee successful job performance, which requires individuals to possess relevant knowledge for task execution, alongside a value system aligned with goal achievement. Motivational factors, associated with expected rewards following task completion, also play a significant role (Schunk & DiBenedetto, 2016). Individuals with high self-efficacy tend to achieve superior performance, exhibit heightened motivation, and experience greater job satisfaction compared to their counterparts. This propensity often drives them to invest additional effort and energy into their work, a critical aspect for healthcare professionals (Abun, 2021; Ahmed et al., 2011).

Self-efficacy in healthcare organizations is linked to various factors, with leadership being a prominent influencer. Leadership, as the process whereby a leader guides employees (followers) towards achieving specific goals through collective effort, is a social phenomenon wherein a leader leverages the resources and capacities of a group (Benmira & Agboola, 2021). However, the effectiveness of leadership and its impact on organizational performance are contingent upon numerous factors, suggesting that there is no singular universally optimal leadership style. At times, leaders are expected to adopt a directive approach, while in other domains they may facilitate decision-making through consensus. Undoubtedly, a leader must strike a balance between organizational objectives and employee needs, demonstrating communicativeness, agility, and adaptability (Waldman et al., 2019). In healthcare organizations, leadership emerges as a critical determinant of healthcare quality. To foster this, leaders within healthcare organizations should cultivate an organizational climate characterized by mutual respect, idea and information exchange, knowledge sharing, participation in problem-solving, active listening, and support for healthcare professionals. Leaders should empower employees in healthcare settings to generate innovative and creative ideas, thereby contributing to organizational success through their expertise (Kline, 2019). Particularly in challenging and evolving circumstances, healthcare leaders should chart a clear path forward, communicate a compelling vision, leverage the expertise of healthcare professionals, drive change, and address resistance to change (Galea, 2017). Given the emphasis on intellectual development and fostering creativity and innovation among employees, specific leadership styles are presumed to be linked to self-efficacy. However, considering that contextual factors influence the effectiveness of leadership styles, it is evident that certain forms of leadership may at times induce stress, potentially impairing the performance of healthcare organizations. By selecting an appropriate leadership style, not only can this improve performance, satisfaction, and motivation, but it can also decrease turnover intention rates (Asif et al., 2019). The way a leader manages and the way group members interact with each other significantly influence stress levels at work. Additionally, the dynamic between leadership and team collaboration plays a crucial role in either reducing or increasing workplace stress (Cho, 2023). Within healthcare organizations, the role and significance of transactional leadership deserve emphasis, given the imperative to optimize healthcare quality and organizational efficiency. Nonetheless, owing to the valuable human capital possessed by healthcare professionals, there is often an assumption that they should be afforded considerable autonomy and latitude, as espoused by laissez-faire leadership. However, it is essential to recognize that such leadership styles can engender several challenges, particularly concerning stress occurrence and the self-efficacy of healthcare professionals.

Workplace stress often proves detrimental to entire organizations, precipitating a decline in productivity and escalating costs. Within healthcare organizations, stress can result in inefficiencies and a deterioration in healthcare quality. Its impact on employees varies depending on personal characteristics and other factors. Mismanaged stress fosters strained interpersonal relationships, emotional tension, and physiological and mental disorders among employees, potentially leading to dire consequences (Iskamto, 2021). Stress manifests as an emotional response to various stressors, which in organizational contexts can be manifold. Given the dynamic nature of stress, stress levels can fluctuate over time, necessitating careful management and identification of contributing factors (Ramlawati et al., 2021). As previously noted, the absence of pertinent information during task execution, along with inadequate leadership support, resources, and clear guidelines, suggests that laissez-faire leadership may exacerbate stress, which in turn can diminish self-efficacy over time (Diebig & Bormann, 2020).

Research carried out on a particular pilot project demonstrated that leaders who participated in the program designed to enhance resilience of healthcare professionals had a beneficial effect on the perceived stress and self-efficacy of staff members (Giordano et al., 2022). Previous studies have demonstrated the varying impacts of different types of leadership on work-related outcomes. A study that involved nursing professionals has confirmed that servant leadership in healthcare settings mitigates workplace stressors, decreases burnout, strengthens job satisfaction, and enhances performance while lowering turnover intentions (Westbrook et al., 2022). The critical role of servant leadership behaviors in supporting employees during challenging times was underscored by the reduction of employee stress levels, the improvement of service quality, and the positive enhancement of motivation during the pandemic (Çağatay et al., 2023). A research conducted in a tertiary hospital in Germany indicated that both transformational and transactional leadership were associated with elevated well-being scores, but laissez-faire leadership was related to diminished scores across nearly all professional groups (Erschens et al., 2022). On the other hand, a cross-sectional study involving nurses from governmental hospitals in Iran revealed a significant negative correlation between transformational and transactional leadership styles and job satisfaction, while a statistically significant positive correlation was found between the laissez-faire leadership style and both job satisfaction and anticipated turnover (Pishgooie et al., 2019). A systematic review of the literature suggested that transformational leadership has substantial positive effects, whereas laissez-faire leadership has a plethora of negative effects on work-related outcomes (Khan & Tidman, 2021). Despite the well-documented adverse impacts of laissez-faire leadership, it remains a relatively underexplored leadership style (Robert & Vandenberghe, 2021). Given the structured, high-pressure, and protocol-driven nature of healthcare, transactional and laissez-faire leadership are often more practical and commonly practiced by healthcare professionals compared to transformational leadership, which requires more time, engagement, and focus on long-term vision (Pishgooie et al., 2019; Xirasagar, 2008). Although servant leadership was associated with favorable results in healthcare, practitioners suggest caution, as integrating these behaviors into healthcare organizations is a challenging endeavor (Demeke et al., 2024). Therefore, our study focuses on transactional and laissez-faire leadership and their relationship with stress and self-efficacy.

Considering the foregoing, this research aims to investigate the influence of leadership on the self-efficacy of healthcare professionals. Additionally, it seeks to explore whether stress mediates the relationship between leadership and self-efficacy. To this end, following the introduction, the second section of the paper entails a literature review, from which research hypotheses are delineated. The third section presents the research findings and subsequent discussion.

## 2. Literature Review

### 2.1. Self-Efficacy

The quality of healthcare delivery and the efficiency of healthcare organizations hinge largely on the knowledge, skills, and behaviors of their employees. The level of engagement and willingness of healthcare professionals to exert additional efforts are influenced by various factors (Kitsios & Kamariotou, 2021). Self-efficacy, as one such factor, embodies an individual’s belief in their own capabilities and potential to control events and functions in their daily life and work. Rooted in self-efficacy theory, it pertains to the conviction that one can mobilize cognitive resources, motivation, and abilities in order to accomplish tasks and achieve set goals (Abun, 2021; Bandura, 1977). Central to this concept is individuals’ perceptions of their abilities and capacities to engage in specific tasks and attain objectives (Waddington, 2023). Social cognitive theory posits that individuals can exert significant control over pivotal events in their lives. Goal-directed behavior and self-efficacy are core components that afford greater control over events, thereby culminating in behaviors conducive to success, motivation, and satisfaction (Schunk & DiBenedetto, 2016). According to this theory, alongside environmental factors, the achievement of specific goals is effectively influenced by individuals’ behaviors and cognitions, including self-efficacy (Gist & Mitchell, 1992). By maintaining certain behaviors, individuals create self-motivators to persist in their efforts until the desired goals or performance standards are achieved. In this context, four pertinent sources of self-efficacy emerge: mastery experiences, vicarious experiences, verbal persuasion, and affective states (Bandura, 1977). Mastery experiences refer to the knowledge and skills individuals gain through the successful execution of prior tasks. Vicarious experiences involve the insights and understanding one acquires by observing others perform tasks. Verbal persuasion encompasses the encouragement and motivation received from others to undertake specific tasks. Psychological and affective states reflect an individual’s belief in their ability to accomplish a task or modify their behavior in order to achieve a desired outcome. These elements collectively contribute to an individual’s self-efficacy and capability in various situations (Waddington, 2023; Schunk & DiBenedetto, 2016). Individuals with high self-efficacy tend to set ambitious goals, actively seek out new knowledge while on the job, and endeavor to tackle challenging tasks (Lunenburg, 2011). Consequently, various types of self-efficacy can be discerned, including self-efficacy for performance, learning, collaborative teaching, and more (Schunk & DiBenedetto, 2016).

Self-efficacy holds immense significance within healthcare organizations, where the quality of healthcare and operational efficiency are directly contingent upon the knowledge and conduct of employees. Healthcare professionals possessing high self-efficacy demonstrate adept problem-solving skills, effectively implement strategies aimed at advancing the missions of healthcare organizations, and exhibit resilience in navigating stressful workplace circumstances (Karagkounis et al., 2020). The self-efficacy of healthcare professionals is shaped by their knowledge, experience, social learning, and interpersonal influences. Consequently, self-efficacy influences healthcare professionals’ willingness to exert additional efforts in delivering quality healthcare, making sound decisions, and adeptly handling various stressors and resource constraints (Mohamed et al., 2023). In addition to its impact on organizational performance, self-efficacy within healthcare organizations fosters heightened motivation, satisfaction, and commitment to achieving additional objectives. Moreover, high self-efficacy correlates with lower levels of burnout among healthcare professionals (Bernales-Turpo et al., 2022). Analogous to an internal locus of control, healthcare professionals’ self-efficacy engenders better control over the work process, leading to enhanced individual outcomes, reduced emotional exhaustion, and overall well-being (Abo-Ali et al., 2021; Najjuka et al., 2022). Furthermore, healthcare professionals with high self-efficacy often demonstrate proactive behaviors, generating creative and innovative ideas that benefit patients and society by elevating the quality of healthcare (Karagkounis et al., 2020).

### 2.2. Leadership

Leadership is conceptualized as the process and art of influencing followers to harness their efforts, skills, knowledge, and experience in order to achieve organizational goals alongside the leader (Specchia et al., 2021). Leaders are tasked with defining goals and vision, articulating reality, effectively communicating messages from top management, and collaborating with employees to accomplish tasks (Ali, 2020). The effectiveness of leadership hinges on employees’ willingness to follow and collaborate with the leader in order to attain specific goals (Iqbal et al., 2021). This, in turn, is influenced by a myriad of contextual factors, necessitating various leadership styles, some of which are particularly pertinent in healthcare organizations. Transformational leadership is often highlighted as paramount in healthcare settings, as leaders motivate, inspire, develop, and leverage the intellect of healthcare professionals. Additionally, leaders serve as role models through their behavior, guiding followers to emulate their actions. However, exigent circumstances may call for a more directive management approach, especially when healthcare professionals are unable to act rationally due to diminished cognitive abilities. Alongside transformational leadership, servant leadership, which focuses on employee development, resonant leadership, which is characterized by high emotional intelligence, and other forms of leadership find application in healthcare organizations (Specchia et al., 2021). Participative leadership, as emphasized by Diggele et al. (2020), entails resolving relevant decisions and complex problems through collaborative discussion and knowledge exchange, with the leader involving the employees in decision-making. Task orientation is also emphasized, albeit not at the expense of healthcare professionals’ needs and attitudes, as an exclusive focus on tasks can strain interpersonal relations and detrimentally affect employee attitudes and behavior, leading to reduced productivity and increased turnover. Consequently, leaders should eschew a top-down decision-making approach and avoid establishing rigid hierarchical relationships with healthcare professionals, embodying instead the principle of primus inter pares. 

Leaders in healthcare organizations are tasked with charting the direction of action, communicating the vision optimistically, fostering teamwork, disseminating relevant information, developing human capital, facilitating change, and engaging the intellect of healthcare professionals during task implementation (Galea, 2017; Kumar & Khiljee, 2016). This necessitates a flexible approach, wherein leaders may alternately adopt transformative, participative, or directive leadership styles, all while prioritizing the overarching goals of maximizing healthcare quality and efficiency. Although healthcare professionals are valued for their knowledge and experience, complete autonomy in their work may not always be conducive to organizational objectives. While participation in leadership processes and autonomy are crucial for motivation and performance, leaders must strike a balance, recognizing that excessive independence can sometimes prove counterproductive. In this regard, the relevant tenets of laissez-faire leadership merit consideration.

In addition to transformational and transactional leadership, laissez-faire leadership emerges as a popular and frequently employed style, as it can foster high levels of innovation and creativity among employees under certain circumstances. Laissez-faire leadership entails the relinquishment of leadership control, granting employees full autonomy in their actions which, in many situations, proves unproductive (Robert & Vandenberghe, 2021). Under laissez-faire leadership, employees enjoy complete decision-making freedom, while the leader refrains from interaction and collaborative problem-solving, relinquishes power and influence, and often shirks responsibility. Consequently, followers often perceive such leaders as ineffective. In this scenario, the leader’s role is minimal or non-existent, leaving employees without the necessary information, resources, and guidelines for work, ultimately resulting in decreased productivity (Thanh & Quang, 2022). According to Avolio (1999), laissez-faire leadership is ineffectual, leading to follower dissatisfaction (Norris et al., 2021). Owing to the absence of control and the extensive freedom granted to employees, laissez-faire leadership precludes performance checks, potentially leading to deteriorating organizational outcomes, which can be very risky in healthcare (Iqbal et al., 2021). Based on the Leader–Member Exchange (LMX) theory, followers may perceive laissez-faire leadership as ineffective due to the lack of support from their leader during work (Robert & Vandenberghe, 2021).

It is crucial to differentiate laissez-faire leadership from delegating responsibility. While delegation involves providing resources, information, deadlines, additional support, performance monitoring, and rewards for achieved results, laissez-faire leadership entails leadership abdication, which is devoid of these elements. Consequently, employees may be uncertain about the rewards for their work, as leaders under laissez-faire leadership often delay action, fail to provide feedback, neglect to acknowledge employee contributions, and ignore their needs. Hence, laissez-faire leadership can be not only ineffective but also detrimental (Robert & Vandenberghe, 2021). Consequently, organizational performance may decline, and employees may become less loyal to the organization (Donkor & Zhou, 2020; Thanh & Quang, 2022). Laissez-faire leadership engenders employee confusion, reduces trust in the leader, and fosters anxiety, significantly diminishing productivity, which is particularly concerning in the healthcare context (Ali, 2020; Specchia et al., 2021). Leader abdication generates uncertainty regarding performance quality, standards, and deadlines, leading to stress among employees. This uncertainty often results in conflicts due to strained emotions and negative mental shifts, exacerbated by the discrepancy between the expected and the actual roles during work. This role ambiguity can be highly stressful for employees, dampening motivation and job satisfaction. The absence of leadership support, in terms of relevant information, resources, and assistance, exacerbates the situation, potentially proving destructive for the entire organization (Diebig & Bormann, 2020; Lundmark et al., 2021). Consequently, laissez-faire leadership contributes to employee stress, thereby reducing productivity, ultimately impacting overall organizational performance (Rowold & Schlotz, 2009). In light of the above, we propose the following research hypothesis:

**H1:**  *Laissez-faire leadership is positively associated with stress.*

In healthcare organizations, the clear communication of duties, obligations, tasks, and goals is essential for healthcare professionals to understand what is expected of them and the extent to which they should utilize their knowledge and skills. Within the framework of self-efficacy, verbal persuasion and psychological states, as outlined by Bandura (1977), emerge as significant sources. Verbal persuasion involves clearly indicating to employees what is required of them, while psychological states entail believing in one’s ability to accomplish a task based on clear information provided beforehand. Therefore, the effective communication of tasks, goals, and feedback plays a pivotal role in determining the level of effort and efficiency achieved by healthcare professionals (Nicola et al., 2020; Liu & Gumah, 2020). In the case of laissez-faire leadership, as repeatedly highlighted, there is a lack of support from the leader during work, resulting in employees being deprived of relevant information (Diebig & Bormann, 2020). Consequently, this leadership style negatively impacts self-efficacy, as evidenced by research conducted by Ridwan et al. (2022). Accordingly, the following research hypothesis can be posited:

**H2:**  *Laissez-faire leadership is negatively associated with self-efficacy.*

In order to deliver effective healthcare, employees must have a clear understanding of their responsibilities. Transactional leadership emphasizes order, structure, formal authority, and goal achievement through an exchange process. The leader delineates goals, communicates expectations, and outlines the rewards employees will receive upon goal attainment (Diebig & Bormann, 2020). A transactional leader endeavors to align organizational goals with the personal goals of employees by recognizing their needs and expectations, clearly communicating rewards for effort, work, and knowledge, and providing these rewards upon task completion and goal achievement (Purwanto et al., 2020; Bass, 1985). Transactional leadership involves the leader recognizing the needs of followers, closely associating them with rewards, monitoring and analyzing performance, and correcting follower actions as necessary to enhance results. However, the leader–follower relationship in transactional leadership should not be construed as authoritarian; rather, it is based on mutual respect and ethical conduct, with the leader trusting in the competence, skills, and experience of followers and delegating tasks accordingly (Specchia et al., 2021). By providing rewards upon meeting or surpassing performance targets, the leader demonstrates trust and appreciation towards followers, affirming the effectiveness of this leadership style (Ali, 2020). In addition to contingent rewards, management by exception entails the leader correcting employee behavior to improve performance. This involves providing additional information, resources, and feedback, as well as enhancing knowledge and skills, thereby enhancing the overall human capital stock, which is conducive to the development of self-efficacy (Thanh & Quang, 2022; Bass, 1985).

Transactional leadership is pragmatic in nature, focusing on the attainment of organizational goals. Leaders often provide extrinsic rewards, although intrinsic rewards, such as additional knowledge, training, and participation in creative tasks are not uncommon (Aarons, 2006). Given that the primary objective of healthcare organizations is to deliver high-quality healthcare, the effectiveness of transactional leadership in healthcare stems from the specific tasks that are necessary to achieve this goal. In healthcare settings, transactional leadership is vital for executing tasks accurately, often entailing contingent rewards (e.g., work resources), provision of relevant information, and management by exception to rectify potential errors and minimize the risk of substandard healthcare delivery (Fletcher et al., 2019). Consequently, transactional leadership positively influences organizational performance (Purwanto et al., 2020; Ali, 2020), as well as the innovation and creativity of organizations (Diggele et al., 2020). Clear expectations and goals under transactional leadership lead to heightened motivation and job satisfaction among employees (Abbas & Ali, 2023; Specchia et al., 2021). The presence of satisfaction and motivation contrasts with stressful circumstances, which are characterized by tension, decreased concentration, emotional strain, conflicts, and subsequent decline in productivity (Yang et al., 2021). Thus, the following research hypothesis can be posited:

**H3:**  *Transactional leadership is negatively associated with stress.*

To foster and leverage the self-efficacy of healthcare professionals, an appropriate leadership style is required, where the leader defines tasks, encourages idea development, provides resources, and offers support, particularly through performance evaluation (Ridwan et al., 2022). Transactional leadership, due to the abundance of information it provides to followers, serves as a means to promote self-efficacy (Beauchamp et al., 2016). Alongside clear work guidelines, transactional leaders provide feedback to employees on the structure and quality of desired performance, offer resource support, and furnish information on task fulfillment levels. Consequently, employees are informed if behavioral adjustments are necessary and to what extent. Moreover, transactional leaders offer various rewards for completed tasks, some of which may be linked to learning and development, which are factors that can bolster self-efficacy (Liu & Gumah, 2020). Consequently, transactional leaders can enhance employees’ creativity levels, which is also a facet of self-efficacy (Wei et al., 2010). Therefore, transactional leadership positively impacts self-efficacy, as evidenced by Ridwan et al. in their research (Ridwan et al., 2022). Thus, the following research hypothesis can be formulated:

**H4:**  *Transactional leadership is positively associated with self-efficacy.*

### 2.3. Stress

The working environment within healthcare organizations is marked by a significant level of stress due to the nature and workload of tasks. Stress levels escalate when employees are confronted with heavy workloads, especially when they lack sufficient resources, knowledge, and support to handle them effectively (Johnson et al., 2020). Joy (2020) and Yang et al. (2021) note that stress typically arises when employees encounter tasks and environments that contradict their values, eliciting specific emotional reactions, such as the need to adapt to challenging circumstances. Stress manifests when employees are expected to fulfill work obligations surpassing their cognitive and mental capacities, or when there is a significant mismatch between task demands and anticipated rewards (Pandey, 2020). Consequently, employees experience anxiety, chronic fatigue, nervousness, and depression, impairing their ability to work efficiently, and potentially leading to various health issues and adverse outcomes (Ramlawati et al., 2021).

Negative emotional and physiological responses to stress hinder employees’ ability to manage daily duties and interpersonal interactions (Sutrisno, 2022). This stress is particularly prevalent and perilous within healthcare organizations, where frontline professionals, such as nurses and technicians, grapple with various challenges and social situations amidst heavy workloads and long hours, exacerbating their emotional well-being. Emotional exhaustion syndrome, depersonalization, and burnout are common issues faced by healthcare professionals under stress (Giuseppe et al., 2021). A comprehensive study conducted in the Italian healthcare sector revealed that nearly 50% of employees had experienced stress, 24.73% exhibited symptoms of depression, and 21.9% reported high stress levels. These employees identified support from their leaders as their most pressing need (Lenzo et al., 2021). Another study by Johnson et al. (Johnson et al., 2020) indicated that healthcare professionals often experience elevated stress levels, resulting in diminished motivation both for themselves and their colleagues. Consequently, stress-related negative emotional and health conditions frequently culminate in employee turnover, exacerbating the shortage of essential human capital necessary for healthcare provision (Benfante et al., 2020; Joy, 2020; Abbas & Ali, 2023). 

Coping with stress within healthcare organizations typically necessitates a high level of self-efficacy, indicating an inverse correlation between the two concepts (Najjuka et al., 2022). Karagkounis et al. (2020) emphasize that employees with high self-efficacy tend to respond better to stress within healthcare settings, underscoring the importance of self-efficacy in promoting well-being and mental resilience among healthcare professionals (Abo-Ali et al., 2021). Conversely, high levels of stress can adversely affect the productivity and behavior of employees with low self-efficacy (Abun, 2021). Employing various support mechanisms and self-regulation techniques can be crucial in reducing work-related stress among healthcare professionals. There are several strategies and interventions that can be implemented both individually and institutionally to enhance preparedness and effectiveness in crisis management (Hijazi et al., 2022). Hence, the following research hypothesis can be posited:

**H5:**  *Stress is negatively associated with self-efficacy.*

The relationship between stress and self-efficacy should also be examined within the context of laissez-faire leadership. Insufficient communication of tasks and expectations in stressful circumstances within healthcare organizations can lead to decreased levels of self-efficacy among professionals, who are unsure of what is expected of them (Joy, 2020). Thus, the following research hypothesis can be proposed:

**H6:**  *Stress mediates the relationship between laissez-faire leadership and self-efficacy.*

Similarly, stress within healthcare organizations may impact the effectiveness of transactional leadership. Stressful circumstances can diminish cognitive abilities and rational thinking, hindering employees’ understanding and interpretation of decisions and tasks (Iskamto, 2021; Pandey, 2020). Employees may become more focused on the source of stress rather than on task completion and the application of knowledge (Sebastian, 2013). Valentina examined the impact of transformational and transactional leadership on employees’ negative behavior, moderated by stress. The findings revealed a negative relationship between stress and transactional leadership, indicating that stress mediated the relationship between transactional leadership and negative behavior, such as loss of task and emotional control, deterioration of social relationships, and tardiness (Valentina, 2021). Accordingly, the following research hypothesis can be proposed:

**H7:**  *Stress mediates the relationship between transactional leadership and self-efficacy*.

## 3. Materials and Methods

### 3.1. Participants and Procedure

To test the hypotheses, a cross-sectional study was conducted, focusing on healthcare professionals, particularly those employed in health organizations in Central Serbia and Belgrade (the capital of Serbia). This research was approved by the Institutional Review Board of the Faculty of Medical Science University of Kragujevac on 27 June 2023 (reference number 01-6581/12-53). This study was conducted in accordance with the principles of the Declaration of Helsinki 1975 (revised in 2013) and its subsequent amendments. To address potential ethical concerns, all participants were informed about the voluntary nature of their participation. This written informed consent was included on the first page of the questionnaire distributed to them, with a section which outlined the academic purposes of the research. The questionnaire, written in Serbian, was distributed electronically between October and December 2023. It did not contain questions related to highly sensitive data to ensure the additional security of the respondents. 

The total sample consisted of 405 respondents, with 395 validly completed questionnaires. Descriptive statistics revealed that the sample included 266 female and 129 male respondents. Regarding age distribution, 118 respondents were under 40 years of age, 126 were between 40 and 50 years of age, and 151 were over 51 years of age. In terms of education, the majority of respondents held a Bachelor’s degree (279 respondents), followed by respondents with a high school diploma (73 respondents), and those respondents with a high school degree (43 respondents). Questions identifying their specific roles within health organizations were not included in the research process.

### 3.2. Measurements

The structured questionnaire used in this research comprised a total of 24 statements and three groups of questions related to gender, age, and education. The variables utilized in this study were derived from findings in the relevant body of literature and adapted to meet the needs of this research in the Serbian language. These statements had been previously validated, and their reliability was confirmed through different studies. Leadership was measured using nine statements from Bass and Avolio (1995, 2000), with five statements assessing transactional leadership and four assessing laissez-faire leadership, available from Mind Garden. Stress was measured using nine statements, based on the research by Cohen et al. (1983). Lastly, self-efficacy was measured using six statements developed by Lazić et al. (2021). Respondents expressed their views on all statements within these variables using a five-point Likert scale.

## 4. Results and Analysis

In order to verify the set research hypotheses, partial least squares structural equation modeling (PLS-SEM) was implemented. Variance-based structural equation modeling (SEM) is a suitable method for complex models and situations where the data deviate from normal distribution, as highlighted by Hair et al. (2014). Given the characteristics of this study, we opted for a variance-based approach utilizing partial least squares SEM (PLS-SEM) instead of the traditional covariance-based SEM. Moreover, the variance-based approach is commonly employed in research studies related to human resource management (Hair et al., 2012; Ringle et al., 2020), further justifying its application in this research. Following the recommendations of Anderson and Gerbing (1988), a two-step approach was employed in our research, first validating the measurement model, and then evaluating the structural model quality. Data preprocessing involved using SPSS v.23 to prepare the indicators for the reflective measurement model before employing SmartPLS 3.0 for subsequent analysis. 

In this study, three constructs were utilized: leadership (as the independent variable), self-efficacy of healthcare professionals (as the dependent variable), and stress (acting as the mediator variable).

### 4.1. Measurement Model Assessment

To evaluate the model’s validity and reliability, confirmatory factor analysis (CFA) was conducted, with the results of internal consistency and validity detailed in Table 1. 

According to Hair et al. (2017), a Composite Reliability (CR) value of at least 0.7 is indicative of an acceptable model. Our findings reveal that all variables exceed this threshold, supporting the model’s acceptability, and demonstrating strong internal consistency. Convergent validity was assessed using the Average Variance Extracted (AVE), which consistently exceeds 0.5 across all scenarios, indicating that the constructs account for over 50% of the variance in the items (Fornell & Larcker, 1981; Chin, 2010). Specifically, AVE values range from 0.620 to 0.645 for all constructs. Collinearity was evaluated using the Variance Inflation Factor (VIF), which consistently remains below 5, signifying the absence of multicollinearity issues.

Discriminant validity was verified employing the heterotrait–monotrait ratio of correlations (HTMT_0.85_) criterion, with detailed outcomes presented in Table 2. 

The results presented in Table 2 indicate that the values are all below 0.640, which is the maximum value in this case. This suggests that the model meets a satisfactory level of discriminant validity (Henseler et al., 2015).

### 4.2. Structural Model Assessment

Before examining the direct effect results in Table 3, it is important to first review the fit indices presented in the second part of the table. Proceeding from the above, structural model analysis was carried out using PLS-SEM with the blindfolding option enabled to assess the predictive quality. The predictive significance of latent variables was assessed using the Stone–Geisser Q^2^ statistic. The calculated values for this index are as follows: 0.086 for self-efficacy and 0.059 for stress. Positive values of the Stone–Geisser Q^2^ indicate the acceptability and quality of the model (Stone, 1974; Geisser, 1974). The coefficient of determination (R^2^) indicates the explanatory power of the model, with values of 13.4% and 7.6% for self-efficacy, suggesting a good level of explanatory capability. The evaluation of model misspecification was conducted using the standardized root mean square residual (SRMR), which should ideally be below 0.08 to avoid misspecification (Henseler et al., 2014; Hu & Bentler, 1998). In the analysis, the SRMR value was found to be 0.061, indicating that the model meets this criterion. Additionally, the goodness-of-fit (GOF) test fell within the acceptable range of 0 to 1, further supporting the adequacy of the model.

To assess the magnitude and significance of the path coefficients, we utilized standard bootstrapping in PLS-SEM. This method was chosen to establish robust confidence intervals (two-sided bias-corrected 95% CIs) for the relationships examined within our hypotheses. Regarding the direct effect, the results presented in Table 3 show that laissez-fair leadership has a statistically significant and positive impact (β = 0.300, *p* < 0.001), as a result of which it can be concluded that the H1 hypothesis is confirmed. However, on the other hand, laissez-fair leadership achieves a negative influence on self-efficacy, but this influence is not statistically significant (β = −0.026, *p* > 0.05). Based on the above result, the H2 hypothesis cannot be accepted. A positive influence, but without statistical significance (β = 0.049, *p* > 0.05) was identified between transactional leadership and stress, on the basis of which the H3 hypothesis cannot be accepted. On the other hand, transactional leadership has a statistically significant and positive influence on self-efficacy (β = 0.274, *p* < 0.001), which is why hypothesis H4 can be accepted. Finally, stress has a negative and statistically significant effect on self-efficacy (β = −0.190, *p* < 0.01), so the H5 hypothesis can be accepted. According to the results, an increase in laissez-fair leadership by one point leads to an increase in stress by 0.300 points. An increase in transactional leadership by one point leads to an increase in self-efficacy by 0.274 points. Finally, an increase in stress by one point leads to a decrease in self-efficacy by 0.190 points.

To test hypotheses H6 and H7, we conducted a standard moderation analysis using SmartPLS. This analysis included bias-corrected bootstrapping with 95% confidence intervals.

Regarding indirect effects, the results shown in Table 4 indicate a statistically significant moderation of stress in the relationship between laissez-fair leadership and self-efficacy (β= −0.057, *p* < 0.01), on the basis of which it can be claimed that the H6 hypothesis is confirmed. On the other hand, the results show that stress does not achieve a statistically significant moderation between transactional leadership and self-efficacy (β= −0.009, *p* > 0.05), as a result of which the H7 hypothesis cannot be confirmed. Considering the obtained direct and indirect effects, the general conclusion is that stress plays a partially statistically significant mediating role between transactional leadership and the self-efficacy of healthcare professionals.

## 5. Discussion

The research results initially indicate a positive correlation between laissez-faire leadership and stress. Laissez-faire leadership in healthcare settings is characterized by leader abdication, often resulting in a lack of responsibility, failure to communicate decisions, and inadequate support for healthcare professionals. While this leadership style may sometimes foster innovation and creativity (Thanh & Quang, 2022), it must be recognized that healthcare organizations prioritize the delivery of high-quality care. Healthcare professionals rely on clear guidance, resources, and support, especially during common stressful situations within healthcare settings (Johnson et al., 2020). These stress-inducing circumstances already challenge productivity, causing anxiety, fear, and other negative emotional changes among healthcare professionals (Lenzo et al., 2021). In such scenarios, employees expect effective communication and feedback from their superiors to mitigate ambiguity and ensure informed decision-making (Nicola et al., 2020; Liu & Gumah, 2020). However, under laissez-faire leadership, such communication is typically lacking, as leaders grant complete autonomy to employees. This deficit in information, resources, and support further exacerbates stress levels, leading to the observed positive association between laissez-faire leadership and stress. Laissez-faire leadership often fails to provide clear direction or expectations to employees. Without clear guidance, employees may struggle to prioritize tasks, leading to increased stress as they attempt to navigate their responsibilities without guidance. Without access to guidance or resources, employees may feel overwhelmed by their workload and uncertain about how to address challenges, contributing to heightened stress levels. In the absence of active leadership involvement, employees may receive minimal feedback on their performance or progress. Without feedback, employees may struggle to gauge their effectiveness or identify areas for improvement, leading to heightened anxiety and stress about their performance. Laissez-faire leadership may be perceived by employees as a lack of concern or interest from their leaders. This perception can foster feelings of insecurity and inadequacy, contributing to stress, as employees grapple with doubts about their value and contributions to the organization. In laissez-faire leadership environments, conflicts may go unaddressed or escalate due to a lack of intervention from leaders. The presence of unresolved conflicts can create a tense and stressful work environment, as employees navigate interpersonal tensions without support or guidance.

Contrary to initial expectations, laissez-faire leadership does not exhibit a negative association with self-efficacy. While there is a negative correlation between these variables, it is not statistically significant. This suggests that although laissez-faire leadership may theoretically diminish self-efficacy, the extent of this effect is not substantial enough to be considered statistically significant. In practice, granting employees a degree of autonomy and freedom, along with leader’s abdication in favor of independent action by healthcare professionals, may actually enhance self-efficacy. This can be attributed to the increased sense of responsibility among employees in such circumstances. Consequently, employees are more inclined to thoroughly analyze situations, evaluate alternatives, and make informed decisions aimed at improving healthcare quality and organizational efficiency. The leader’s adoption of a laissez-faire approach may stem from a belief in employees’ competencies, knowledge, skills, and self-confidence, thus affording employees the opportunity to exercise independent decision-making. In their pursuit to minimize errors and incorrect decisions, healthcare professionals endeavor to leverage their competencies to the fullest extent possible. Despite the drawbacks of laissez-faire leadership, one potential benefit is the autonomy it affords to employees. In some cases, employees may thrive when given the freedom to make their own decisions and work independently. This autonomy can foster feelings of empowerment and self-efficacy, as employees gain confidence in their ability to manage their responsibilities effectively. Laissez-faire leadership may create opportunities for employees to take on new challenges and develop their skills. When given the freedom to explore different approaches and solutions, employees may experience personal and professional growth, leading to increased self-efficacy as they expand their capabilities and knowledge. Self-efficacy is influenced by various individual factors, including personality traits, past experiences, and cognitive abilities. While laissez-faire leadership may have a negative impact on some employees’ self-efficacy, others may be less affected or even thrive under this leadership style, depending on their unique characteristics and circumstances. The relationship between leadership style and self-efficacy may be influenced by contextual factors within the organization, such as the nature of the work, organizational culture, and the supportiveness of coworkers. These contextual factors can mitigate or exacerbate the effects of laissez-faire leadership on self-efficacy, making it difficult to generalize the relationship across all situations. Hence, these factors require additional research into the factors that influence the self-efficacy of healthcare professionals.

The analysis results revealed that transactional leadership is not negatively associated with stress, contrary to initial expectations. Transactional leadership is characterized by the establishment of guidelines, procedures, goals, and work rules, among other directives. Additionally, transactional leaders provide resources, support, and performance correction when necessary, in order to minimize operational errors, which is a critical aspect within healthcare organizations. While this leadership style offers rewards for achieving goals, it also entails potential penalties for failure to meet the goals, which could contribute to stress. Healthcare professionals, striving to maximize performance and avoid errors, may exert efforts and energy beyond their capacities. Consequently, the presence of tension could induce various emotional states associated with stress among healthcare professionals, posing a threat to their productivity and potentially leading to poorer performance and repercussions. Transactional leaders in healthcare organizations often set high-performance expectations for their employees, which can create pressure and stress to meet those standards. Employees may feel stressed when they perceive that failure to meet these expectations could lead to negative consequences or missed opportunities for rewards. In transactional leadership, rewards are often contingent on meeting specific performance criteria. This can lead to stress among employees, who feel pressured to perform at a certain level to facilitate receiving rewards or avoid penalties, especially if they perceive the rewards as insufficient or unfair. Transactional leaders tend to provide clear instructions and guidelines for tasks, leaving little room for autonomy or creativity. Employees who prefer autonomy in their work may experience stress when they feel constrained by rigid directives or lack opportunities for self-expression. This is often very important for healthcare professionals. If transactional leaders react to stress by increasing control or imposing stricter performance measures, it can exacerbate stress levels among employees. This response may create a cycle where stress leads to more control, which in turn increases stress further.

However, precisely because the leader defines rules and guidelines and provides support, resources, and other structures, there is a connection between transactional leadership and self-efficacy. Leaders strive to make the best use of the competencies and skills possessed by healthcare professionals, resulting in the development of additional self-confidence and motivation to engage their own cognitive capacities. However, in the presence of stress, self-efficacy can decrease. The reason is the fact that stress reduces the cognitive abilities of employees and thereby reduces the possibility of developing and applying the acquired knowledge. In stressful circumstances, there is a greater emphasis on stressors than on one’s own work, due to the need for the organism to adapt to new circumstances (Abun, 2021). The obtained result is as expected and in accordance with the results obtained in the research by Najjuka et al. (2022). Transactional leaders often provide clear instructions, expectations, and goals to their employees. When employees understand what is expected of them and how their performance will be evaluated, they may feel more confident in their ability to meet those expectations, thus enhancing their self-efficacy. Transactional leaders typically provide regular feedback on employees’ performance, highlighting areas of strength and areas needing improvement. Constructive feedback can help employees develop a more accurate perception of their abilities and skills, which can contribute to higher levels of self-efficacy. When employees receive tangible rewards, such as bonuses, promotions, or recognition for their achievements, it can bolster their confidence in their ability to perform well and succeed in their roles. Transactional leaders often establish clear structures and processes within the organization. A structured work environment can provide employees with a sense of stability and predictability, which can enhance their feelings of competence and self-assurance in their ability to navigate their tasks effectively.

Stress mediates the relationship between laissez-faire leadership and self-efficacy. As previously stated, laissez-faire leadership and self-efficacy are in a negative relationship, but not to an extent that can be considered statistically significant. Therefore, in some circumstances, giving freedom to healthcare professionals can lead to an increase in self-efficacy due to the possibility to implement their acquired human capital. Thanh and Quang (2022) note that laissez-faire leadership can be beneficial for innovation and creativity in some circumstances. However, if stress is included between these phenomena, changes occur. Namely, stress appears as a factor that is positively related to laissez-faire leadership but also as a factor that has a negative effect on self-efficacy. Therefore, when healthcare professionals find themselves under stress, there may be a decrease in self-efficacy, which, in such circumstances, requires more detailed information and feedback to ensure effective action. This, once again, requires an adequate role of the leader, who will provide the necessary support during work, which is not the case with laissez-faire leadership. Due to the pronounced positive correlation between transactional leadership and self-efficacy, it cannot be said that stress mediates the relationship between transactional leadership and self-efficacy. Rather, it just confirms what was mentioned previously, namely that in stressful circumstances, the leader is required to provide additional support to the employees. Apart from resources, this support requires communication of all relevant information, goals, procedures, as well as feedback. The effect of stress in such circumstances is much smaller, and this shows that transactional leadership is one of the ways to manage stress in healthcare organizations, which can be beneficial to preserve self-efficacy.

## 6. Limitations and Recommendations for Future Research

The work has certain limitations, which are also guidelines for future research. First, due to the self-report nature of the questionnaire used in this study, there is a possibility that participants may have responded in a socially desirable manner, potentially introducing bias into the sample. Several hypotheses were validated in this study, indicating significant findings within the scope of our research. However, it is noteworthy that our research design relied on cross-sectional data. Cross-sectional studies, while valuable for capturing a snapshot of the data at a single point in time, inherently limit our ability to establish causal relationships between variables. They provide insights into associations and correlations rather than causation. To ascertain causality, longitudinal or experimental designs that track changes over time are typically more appropriate. Therefore, while our study contributes valuable insights, further research using different methodologies would be necessary to establish causal relationships definitively. Second, due to time and resource constraints, our findings are limited to specific regions within Serbia. To enhance the generalizability of our results, future research could broaden the scope of data collection to encompass a more diverse geographic representation. This expansion would allow for a more comprehensive understanding of the broader population and improve the applicability of the findings. Since the empirical findings indicate the absence of mediation effects of stress between transactional leadership and self-efficacy, these results suggest the presence of other potential variables influencing this relationship. Future research could explore alternative mediation variables from various theoretical perspectives to enrich the discussion and deepen the understanding of the problem. This approach would contribute to a more comprehensive exploration of the factors that may influence the relationship between leadership and self-efficacy in different organizational contexts, including organizational culture and resource availability. Future studies could also explore the influence of sociodemographic variables and employee characteristics on the observed effects. Additionally, investigating how various characteristics of subordinates moderate the impact of leadership styles could be a pertinent area of research. This approach would provide valuable insights into how factors such as age, gender, education level, and other sociodemographic attributes interact with leadership styles, thereby enriching our understanding of effective leadership practices across diverse organizational contexts and their impact on stress and self-efficacy. Third, it would be beneficial to investigate organizational characteristics, such as strategy, supervision practices, and organizational values, as potential mediators in the relationship between leadership and self-efficacy. This approach would contribute to a more holistic understanding of the mechanisms through which leadership practices influence healthcare workplace outcomes. Lastly, although validated scales were employed in this study to observe variables, their choice is not necessarily predicted in healthcare settings, which could potentially represent a limitation of this study. Applying scales that can more effectively capture the intricacies of the relationship between leadership style and stress in healthcare settings, including transformational and servant leadership and comparison to laissez-faire leadership or transactional leadership, might contribute to addressing this limitation.

## 7. Conclusions

The exploration of leadership styles, stress, and self-efficacy within healthcare organizations reveals complex relationships. Laissez-faire leadership, characterized by leader abdication and minimal guidance, emerges as a significant predictor of heightened stress among healthcare professionals. This failure of this leadership style to provide essential support and clear communication increases stress levels, undermining employee morale and potentially compromising patient care quality. Contrary to expectations, laissez-faire leadership does not significantly diminish self-efficacy among healthcare professionals. Instead, it occasionally fosters autonomy and responsibility, thereby enhancing self-efficacy in situations where employees thrive on independence. However, the absence of supportive leadership practices under laissez-faire conditions underscores the need for effective guidance and feedback to maintain high levels of self-efficacy and job satisfaction. In contrast, transactional leadership, characterized by clear goals, structured feedback, and rewards tied to performance, demonstrates a positive correlation with self-efficacy. Despite setting high expectations that may induce stress, transactional leaders mitigate these effects through supportive measures and constructive feedback, thereby increasing employees’ confidence in their abilities and promoting professional growth. Stress mediates the relationship between laissez-faire leadership and self-efficacy negatively, highlighting the detrimental impact of inadequate leadership support on employee effectiveness and well-being. In contrast, transactional leadership’s structured approach diminishes the adverse effects of stress on self-efficacy, emphasizing the importance of supportive leadership in mitigating workplace stressors. Overall, these findings underscore the critical role of leadership in shaping organizational culture and employee outcomes within healthcare organizations. Effective leadership practices that prioritize clear communication, supportive feedback mechanisms, and strategic resource allocation are essential for fostering a resilient workforce capable of delivering high-quality patient care amidst the challenges of modern healthcare settings.

## Figures and Tables

**Table 1:** Measurement model and constructs.

Construct and Items	Loadings	VIF	CR	α	AVE
TL: Transactional leadership			0.884	0.864	0.645
TL01	0.821	1.903			
TL02	0.800	1.747			
TL03	0.803	2.240			
TL04	0.735	1.716			
TL05	0.851	2.217			
LFL: Laissez-faire leadership			0.820	0.798	0.620
LFL01	0.833	1.640			
LFL02	0.800	1.713			
LFL03	0.730	1.593			
LFL04	0.783	1.582			
ST: Stress			0.943	0.928	0.632
ST01	0.782	2.234			
ST02	0.741	2.142			
ST03	0.728	2.125			
ST04	0.775	2.214			
ST05	0.794	2.353			
ST06	0.800	2.164			
ST07	0.877	3.413			
ST08	0.800	2.392			
ST09	0.848	2.797			
SE: Self-efficacy			0.903	0.880	0.623
SE01	0.824	2.197			
SE02	0.850	2.561			
SE03	0.741	1.891			
SE04	0.862	2.665			
SE05	0.740	2.088			
SE06	0.705	1.587			

**Table 2:** Discriminant validity (HTMT_0.85_ criterion).

Constructs	1	2	3	4
1. LFL: Laissez-faire leadership	–			
2. SE: Self-efficacy	0.251			
3. ST: Stress	0.288	0.233		
4. TL: Transactional leadership	0.640	0.325	0.126	–

**Table 3:** Results of testing the hypothesis: direct effects.

Relationship	Path Coefficient		*t*-Value	95% CIs (Bias Corrected)	Hypothesis	Results
LFL → ST	0.300 ***		4.909	[0.166, 0.409]	H1	Supported
LFL → SE	−0.026		0.467	[−0.130, 0.083]	H2	Not supported
TL → ST	0.049		0.744	[−0.076, 0.184]	H3	Not supported
TL → SE	0.274 ***		5.404	[0.162, 0.365]	H4	Supported
ST → SE	−0.190 **		3.475	[−0.285, −0.069]	H5	Supported
Stoner-Geisser Q^2^		R^2^	GOF			
Self-efficacy	0.086	0.134	0.107			
Stress	0.059	0.076	0.067			
SRMR	0.061					

Notes: TL, transactional leadership; LFL, laissez-faire leadership; ST, stress; SE, self-efficacy. ** *p* < 0.01; *** *p* < 0.001.

**Table 4:** Results of testing the hypothesis: indirect effects.

Relationship	Path Coefficient	*t*-Value	95% CIs (Bias Corrected)	Hypothesis	Results
LFL → ST → SE	−0.057 **	2.718	[−0.100, −0.020]	H6	Supported
TL → ST → SE	−0.009	0.687	[−0.041, 0.014]	H7	Not supported

Notes: TL, transactional leadership; LFL, laissez-faire leadership; ST, stress; SE, self-efficacy. ** *p* < 0.01.

## Data Availability

The data presented in this study are available on request from the corresponding author due to ethical reasons.

## References

[B1-behavsci-15-00025] Aarons G. A. (2006). Transformational and transactional leadership: Association with attitudes toward evidence-based practice. Psychiatric Services.

[B2-behavsci-15-00025] Abbas M., Ali R. (2023). Transformational versus transactional leadership styles and project success: A meta-analytic review. European Management Journal.

[B3-behavsci-15-00025] Abo-Ali E. A., Al-Rubaki S., Lubbad S., Nchoukati M., Alqahtani R., Albraim S., Ghareeb W. A., Al-Haffashi B., Alghamdi F., Zaytoun S. (2021). Mental well-being and self-efficacy of healthcare workers in Saudi Arabia during the COVID-19 pandemic. Risk Management and Healthcare Policy.

[B4-behavsci-15-00025] Abun D. (2021). Employees’ self-efficacy and work performance of employees as mediated by work environment. International Journal of Research in Business and Social Science.

[B5-behavsci-15-00025] Ahmed I., Qazi T. F., Jabeen S. (2011). Self-efficacy: The predictor of educational performance among university students. Information Management and Business Review.

[B6-behavsci-15-00025] Ali R. (2020). Exploring the benefits and limitations of transactional leadership in healthcare. Nursing Standard.

[B7-behavsci-15-00025] Anderson J. C., Gerbing D. W. (1988). Structural equation modeling in practice: A review and recommended two-step approach. Psychological Bulletin.

[B8-behavsci-15-00025] Asif M., Jameel A., Hussain A., Hwang J., Sahito N. (2019). Linking transformational leadership with nurse-assessed adverse patient outcomes and the quality of care: Assessing the role of job satisfaction and structural empowerment. International Journal of Environmental Research and Public Health.

[B9-behavsci-15-00025] Avolio B. J. (1999). Full leadership development: Building the vital forces in organizations.

[B10-behavsci-15-00025] Bandura A. (1977). Self-efficacy: Toward a unifying theory of behavioral change. Psychological Review.

[B11-behavsci-15-00025] Bass B. M. (1985). Leadership and performance beyond expectations.

[B12-behavsci-15-00025] Bass B. M., Avolio B. J. (1995). MLQ multifactor leadership questionnaire for research: Permission Set.

[B13-behavsci-15-00025] Bass B. M., Avolio B. J. (2000). MLQ: Multifactor leadership questionnaire: Third edition manual and sampler set.

[B14-behavsci-15-00025] Beauchamp M. R., Welch A. S., Hulley A. J. (2016). Transformational and transactional leadership and exercise-related self-efficacy. Journal of Health Psychology.

[B15-behavsci-15-00025] Benfante A., Di Tella M., Romeo A., Castelli L. (2020). Traumatic stress in healthcare workers during the COVID-19 pandemic: A review of the immediate impact. Frontiers in Psychology.

[B16-behavsci-15-00025] Benmira S., Agboola M. (2021). Evolution of leadership theory. BMJ Leader.

[B17-behavsci-15-00025] Bernales-Turpo D., Quispe-Velasquez R., Flores-Ticona D., Saintila J., Ruiz Mamani P. G., Huancahuire-Vega S., Morales-Garcia M., Morales-Garcia W. C. (2022). Burnout, professional self-efficacy, and life satisfaction as predictors of job performance in health care workers: The mediating role of work engagement. Journal of Primary Care and Community Health.

[B18-behavsci-15-00025] Chin W. W., Vinzi V. E., Chin W. W., Henseler J., Wang H. (2010). How to write up and report PLS analysis. Handbook of partial least squares.

[B19-behavsci-15-00025] Cho C. C. (2023). A cross-level study of the consequences of work stress in police officers: Using transformational leadership and group member interactions as an example. Psychology Research and Behavior Management.

[B20-behavsci-15-00025] Cohen S., Kamarck T., Mermelstein R. (1983). A global measure of perceived stress. Journal of Health and Social Behavior.

[B21-behavsci-15-00025] Çağatay A., Topal A., Arslan Ü. (2023). The Effect Of Perceived Stress of Health Care Professionals on Servant Leadership Behaviors In The Covid-19 Period: NMRT Example. International Journal of Health Management and Tourism.

[B22-behavsci-15-00025] Demeke G. W., van Engen M. L., Markos S. (2024). Servant leadership in the healthcare literature: A systematic review. Journal of Healthcare Leadership.

[B23-behavsci-15-00025] Diebig M., Bormann K. C. (2020). The dynamic relationship between laissez-faire leadership and day-level stress: A role theory perspective. German Journal of Human Resource Management.

[B24-behavsci-15-00025] Diggele C., Burgess A., Roberts C., Mellis C. (2020). Leadership in healthcare education. BMC Medical Education.

[B25-behavsci-15-00025] Donkor F., Zhou D. (2020). Organisational commitment influences on the relationship between transactional and laissez-faire leadership styles and employee performance in the Ghanaian public service environment. Journal of Psychology in Africa.

[B26-behavsci-15-00025] Erschens R., Seifried-Dübon T., Stuber F., Rieger M. A., Zipfel S., Nikendei C., Genrich M., Angerer P., Maatouk I., Gündel H., Rothermund E., Peters M., Junne F. (2022). The association of perceived leadership style and subjective well-being of employees in a tertiary hospital in Germany. PLoS ONE.

[B27-behavsci-15-00025] Fletcher K. A., Friedman A., Piedimonte G. (2019). Transformational and transactional leadership in healthcare seen through the lens of pediatrics. Journal of Pediatrics.

[B28-behavsci-15-00025] Fornell C., Larcker D. F. (1981). Evaluating structural equation models with unobservable variables and measurement error. Journal of Marketing Research.

[B29-behavsci-15-00025] Galea M. (2017). Applying leadership styles to the healthcare sector. Journal of the Malta College of Family Doctors.

[B30-behavsci-15-00025] Geisser S. (1974). A predictive approach to the random effects model. Biometrika.

[B31-behavsci-15-00025] Giordano F., Cipolla A., Ungar M. (2022). Building resilience for healthcare professionals working in an Italian red zone during the COVID-19 outbreak: A pilot study. Stress and Health.

[B32-behavsci-15-00025] Gist M. E., Mitchell T. R. (1992). Self-efficacy: A theoretical analysis of its determinants and malleability. Academy of Management Review.

[B33-behavsci-15-00025] Giuseppe M., Nepa G., Prout T. A., Albertini F., Marcelli S., Orru G., Conversano C. (2021). Stress, burnout, and resilience among healthcare workers during the COVID-19 emergency: The role of defense mechanisms. International Journal of Environmental Research and Public Health.

[B34-behavsci-15-00025] Hair J. F., Hollingsworth C. L., Randolph A. B., Chong A. Y. (2017). An updated and expanded assessment of PLS-SEM in information systems research. Industrial Management and Data Systems.

[B35-behavsci-15-00025] Hair J. F., Sarstedt M., Hopkins L., Kuppelwieser V. G. (2014). Partial least squares structural equation modeling (PLS-SEM): An emerging tool in business research. European Business Review.

[B36-behavsci-15-00025] Hair J. F., Sarstedt M., Pieper T. M., Ringle C. M. (2012). The use of partial least squares structural equation modeling in strategic management research: A review of past practices and recommendations for future applications. Long Range Planning.

[B37-behavsci-15-00025] Henseler J., Dijkstra T. K., Sarstedt M., Ringle C. M., Diamantopoulos A., Straub D. W., Ketchen D. J., Hair J. F., Hult G. T. M., Calantone R. J. (2014). Common beliefs and reality about PLS: Comments on Rönkkö and Evermann (2013). Organizational Research Methods.

[B38-behavsci-15-00025] Henseler J., Ringle C. M., Sarstedt M. (2015). A new criterion for assessing discriminant validity in variance-based structural equation modeling. Journal of the Academy of Marketing Science.

[B39-behavsci-15-00025] Hijazi H., Baniissa W., Abdi R. A. (2022). Experiences of work-related stress among female healthcare workers during the COVID-19 public health emergency: A qualitative study in the United Arab Emirates. Psychology Research and Behavior Management.

[B40-behavsci-15-00025] Hu L. T., Bentler P. M. (1998). Fit indices in covariance structure modeling: Sensitivity to underparameterized model misspecification. Psychological Methods.

[B41-behavsci-15-00025] Iqbal A. Z., Abid G., Arshad M., Ashfaq F., Athar M. A., Hassan Q. (2021). Impact of authoritative and laissez-faire leadership on thriving at work: The moderating role of conscientiousness. European Journal of Investigation in Health, Psychology, and Education.

[B42-behavsci-15-00025] Iskamto D. (2021). Stress and its impact on employee performance. International Journal of Social and Management Studies.

[B43-behavsci-15-00025] Johnson A. R., Jayappa R., James M. (2020). Do low self-esteem and high stress lead to burnout among health-care workers? Evidence from a tertiary hospital in Bangalore, India. Safety and Health at Work.

[B44-behavsci-15-00025] Joy H. (2020). Stress management and employee performance. European Journal of Human Resource Management Studies.

[B45-behavsci-15-00025] Karagkounis C., Manomenidis G., Platis C. G., Minasidou E., Bellali T. (2020). The impact of self-efficacy and work engagement on healthcare professionals’ proactive behavior. Health.

[B46-behavsci-15-00025] Khan A., Tidman M. (2021). Impacts of transformational and Laissez-Faire leadership in health. International Journal of Medical Science and Clinical Invention.

[B47-behavsci-15-00025] Kitsios F., Kamariotou M. (2021). Job satisfaction behind motivation: An empirical study in public health workers. Heliyon.

[B48-behavsci-15-00025] Kline R. (2019). Leadership in the NHS. BMJ Leader.

[B49-behavsci-15-00025] Kumar R. D. C., Khiljee N. (2016). Leadership in healthcare. Anaesthesia and Intensive Care Medicine.

[B50-behavsci-15-00025] Lazić M., Jovanović V., Gavrilov-Jerković V. (2021). The general self-efficacy scale: New evidence of structural validity, measurement invariance, and predictive properties in relationship to subjective well-being in Serbian samples. Current Psychology.

[B51-behavsci-15-00025] Lenzo V., Quattropani M. C., Sardella A., Martino G., Bonanno G. A. (2021). Depression, anxiety, and stress among healthcare workers during the COVID-19 outbreak and relationships with expressive flexibility and context sensitivity. Frontiers in Psychology.

[B52-behavsci-15-00025] Liu W., Gumah B. (2020). Leadership style and self-efficacy: The influences of feedback. Journal of Psychology in Africa.

[B53-behavsci-15-00025] Lundmark R., Richter A., Tafvelin S. (2021). Consequences of managers’ laissez-faire leadership during organizational restructuring. Journal of Change Management.

[B54-behavsci-15-00025] Lunenburg F. C. (2011). Self-efficacy in the workplace: Implications for motivation and performance. International Journal of Management, Business, and Administration.

[B55-behavsci-15-00025] Mohamed O. M. E., Malumoud H. G., El-Sabahy H. E. (2023). Role of self-efficacy and work resilience in head nurses’ job insecurity at Main Mansoura University Hospital. Mansoura Nursing Journal.

[B56-behavsci-15-00025] Najjuka S. M., Ngabirano T. D., Balizzakiwa T., Nabadda R., Kaggwa M. M., Kateete D. P., Kalungi S., Beyeza-Kashesya J., Kiguli S. (2022). Health care workers’ perceived self-efficacy to manage COVID-19 patients in Central Uganda: A cross-sectional study. Risk Management and Healthcare Policy.

[B57-behavsci-15-00025] Nicola A. B., Sohrabi C. D., Mathew G. (2020). Health policy and leadership models during the COVID-19 pandemic: A review. International Journal of Surgery.

[B58-behavsci-15-00025] Norris K. R., Ghahremani H., Lemoine G. J. (2021). Is it laissez-faire leadership or delegation? A deeper examination of an oversimplified leadership phenomenon. Journal of Leadership and Organizational Studies.

[B59-behavsci-15-00025] Pandey D. L. (2020). Work stress and employee performance: An assessment of the impact of work stress. International Research Journal of Human Resources and Social Sciences.

[B60-behavsci-15-00025] Pishgooie A. H., Atashzadeh-Shoorideh F., Falcó-Pegueroles A., Lotfi Z. (2019). Correlation between nursing managers’ leadership styles and nurses’ job stress and anticipated turnover. Journal of Nursing Management.

[B61-behavsci-15-00025] Purwanto A., Bernarto I., Asbari M., Wijayanti L. M., Hyun C. C. (2020). Effect of transformational and transactional leadership style on public health centre performance. Journal of Research in Business and Economics Education.

[B62-behavsci-15-00025] Ramlawati R., Trisnawati E., Yasin N. A., Kurniawaty K. (2021). External alternatives, job stress on job satisfaction and employee turnover intention. Management Science Letters.

[B63-behavsci-15-00025] Ridwan R., Sudjarwo S., Sulpakar S. (2022). The effects of transformational, transactional, and laissez-faire leadership on principals’ self-efficacy. WSEAS Transactions on Advances in Engineering Education.

[B64-behavsci-15-00025] Ringle C. M., Sarstedt M., Mitchell R., Gudergan S. P. (2020). Partial least squares structural equation modeling in HRM research. International Journal of Human Resource Management.

[B65-behavsci-15-00025] Robert V., Vandenberghe C. (2021). Laissez-faire leadership and affective commitment: The roles of leader-member exchange and subordinate relational self-concept. Journal of Business and Psychology.

[B66-behavsci-15-00025] Rowold J., Schlotz W. (2009). Transformational and transactional leadership and followers’ chronic stress. Leadership Review.

[B67-behavsci-15-00025] Schunk D. H., DiBenedetto M. K. (2016). Self-efficacy theory in education. Handbook of motivation at school.

[B68-behavsci-15-00025] Sebastian V. (2013). A theoretical approach to stress and self-efficacy. Procedia-Social and Behavioral Sciences.

[B69-behavsci-15-00025] Specchia M. L., Cozzolino M. R., Carini E., Di Pilla A., Galletti C., Ricciardi W., Damiani G. (2021). Leadership styles and nurses’ job satisfaction: Results of a systematic review. International Journal of Environmental Research and Public Health.

[B70-behavsci-15-00025] Stone M. (1974). Cross-validatory choice and assessment of statistical predictions. Journal of the Royal Statistical Society.

[B71-behavsci-15-00025] Sutrisno S. (2022). Determinants of employee performance: Overview of aspects of communication, work stress, and compensation. Budapest International Research and Critics Institute Journal.

[B72-behavsci-15-00025] Thanh N. H., Quang N. V. (2022). Transformational, transactional, laissez-faire leadership styles and employee engagement: Evidence from Vietnam’s public sector. SAGE Open.

[B73-behavsci-15-00025] Valentina H. V. (2021). The effect of transformational and transactional leadership on employee negative behavior mediated by work stress. International Journal of Science, Technology, and Management.

[B74-behavsci-15-00025] Waddington J. (2023). Self-efficacy. ELT Journal.

[B75-behavsci-15-00025] Waldman D. A., Siegel D. S., Stahl G. K. (2019). Defining the socially responsible leader: Revisiting issues in responsible leadership. Journal of Leadership and Organizational Studies.

[B76-behavsci-15-00025] Wei F., Yuan X., Di Y. (2010). Effects of transactional leadership, psychological empowerment, and empowerment climate on creative performance of subordinates: A cross-level study. Frontiers of Business Research in China.

[B77-behavsci-15-00025] Westbrook K. W., Nicol D., Nicol J. K., Orr D. T. (2022). Effects of servant leadership style on hindrance stressors, burnout, job satisfaction, turnover intentions, and individual performance in a nursing unit. Journal of Health Management.

[B78-behavsci-15-00025] Xirasagar S. (2008). Transformational, transactional and laissez-faire leadership among physician executives. Journal of Health Organization and Management.

[B79-behavsci-15-00025] Yang S. Y., Chen S. C., Lee L., Liu Y. S. (2021). Employee stress, job satisfaction, and job performance: A comparison between high-technology and traditional industry in Taiwan. Journal of Asian Finance, Economics, and Business.

